# Effects of L-glutamine supplementation on degradation rate and rumen fermentation characteristics *in vitro*

**DOI:** 10.5713/ab.21.0279

**Published:** 2021-09-15

**Authors:** Jung-Keun Suh, Jalil Ghassemi Nejad, Yoon-Seok Lee, Hong-Sik Kong, Jae-Sung Lee, Hong-Gu Lee

**Affiliations:** 1 Department of Animal Science and Technology, Sanghuh College of Life Sciences, Konkuk University, Seoul 05029, Korea; 2 Department of Biotechnology, College of Agriculture and Life Science, Hankyong National University, Anseong 17579, Korea; 3 Gyeonggi Regional Research Center, Hankyong National University, Anseong 17579, Korea

**Keywords:** L-glutamine, Degradability of Nutrients, Degradation Rate, Gas Production, *In-vitro* Fermentation

## Abstract

**Objective:**

Two follow-up studies (exp. 1 and 2) were conducted to determine the effects of L-glutamine (L-Gln) supplementation on degradation and rumen fermentation characteristics *in vitro*.

**Methods:**

First, rumen liquor from three cannulated cows was used to test L-Gln (50 mM) degradation rate and ammonia-N production at 6, 12, 24, 36, and 48 h after incubation (exp. 1). Second, rumen liquor from two cannulated steers was used to assess the effects of five levels of L-Gln including 0% (control), 0.5%, 1%, 2%, and 3% at 0, 3, 6, 12, 24, 36, and 48 h after incubation on fermentation characteristics, gas production, and degradability of nutrients (exp. 2).

**Results:**

In exp. 1, L-Gln degradation rate and ammonia-N concentrations increased over time (p<0.001). In exp. 2, pH was reduced significantly as incubation time elapsed (p<0.001). Total gas production tended to increase in all groups as incubation time increased. Acetate and propionate tended to increase by increasing glutamine (Gln) levels, whereas levels of total volatile fatty acids (VFAs) were the highest in 0.5% and 3% Gln groups (p<0.001). The branched-chain VFA showed both linear and quadratic effects showing the lowest values in the 1% Gln group particularly after 6 h incubation (p<0.001). L-Gln increased crude protein degradability (p<0.001), showing the highest degradability in the 0.5% Gln group regardless of incubation time (p<0.05). Degradability of acid detergent fiber and neutral detergent fiber showed a similar pattern showing the highest values in 0.5% Gln group (p<0.10).

**Conclusion:**

Although L-Gln showed no toxicity when it was supplemented at high dosages (2% to 3% of DM), 0.5% L-Gln demonstrated the positive effects on main factors including VFAs production *in-vitro*. The results of this study need to be verified in further *in-vivo* study.

## INTRODUCTION

Glutamine (Gln), a conditionally essential amino acid (AA) in animals [1,2], is the most abundant free AA in the body [2]. It encompasses about 60% of free AA in skeletal cells [3,4] and 20% in plasma [5]. Furthermore, L-Gln has a wide range of cellular and physiological functions including protein synthesis [6], lipid metabolism, and cell growth [7,8], all of which are associated with muscle growth and proliferation [9]. By inducing lymphocyte proliferation, Gln is considered as booster of immune system. Gln also is important in control of metabolic acidosis. Gln involves in the nucleic acid biosynthesis that is necessary to support cell proliferation [10]. Gln may provide a protective effect against hepatic AA oxidation, particularly for methionine (Met) that showed the anabolic potential of Gln since the Met is considered as the first limiting AA in many of ruminant’s feeds [10]. Besides having many proposed metabolic roles, responses to Gln supplementation have been reportedly inconsistent whereas both positive and null effects on production responses have been reported [10]. Understanding the optimum level of Gln supplementation by performing *in-vitro* study will assist to make a better decision to maximizing the muscle growth in empirical *in-vivo* studies in beef cattle while help avoiding possible toxic level(s). The effects of AA supplementation on the production of volatile fatty acids (VFAs) triggered by ruminal microorganisms that ultimately can affect cell growth and production performance is of importance [11,12]. Warner [13] stated that microorganisms from the rumen of sheep rapidly break down Gln to other components such as asparagine and nicotinamide resulting in exceeding ammonia production which in turn can lead to Gln synthesis. This phenomenon implies that although some AAs such as Gln (and asparagine) are more likely to rapidly break down by rumen microorganism to ammonia and microbial protein [13], they are the first to be anabolized or derived from the microbial protein in forestomach by utilizing ammonia [14,15] or in latter gastrointestinal tract (i.e. duodenum) and later responsible for over 50% of AAs entering to the blood stream [16]. Lately, Apajalahti et al [17] have reported positive effects of branched chained AAs, which have different metabolism pathways than Gln, on the yield of branched chained VFA that can be used to estimate the degree of protein degradation. Ruminants have capacity of the forestomach mucosa to utilize ammonia to synthesize Gln and glutamate (Glu) [14,15]. Given this and due to the enormous role of Gln on protein synthesis, cellular and microorganism growth and development, we hypothesized that L-Gln supplementation has potential to positively effect rumen fermentation characteristics in ruminants that need to be first tested *in vitro*. Although recent studies have determined effects of supplementation of valine, leucine, and isoleucine on pH, ammonia-N, branched-chained VFA [11,17], and nutrient digestibility *in-vitro* [18], *in-vitro* effects of L-Gln supplementation at various levels with different incubation time on substrate degradation and fermentation characteristics have not been examined. Thus, the objectives of this study were: i) to determine effects of L-Gln supplementation on its *in-vitro* degradation rate and ammonia-N production; ii) to determine the optimum level(s) of L-Gln supplementation without toxic effects; and iii) to investigate the effects of L-Gln supplementation on fermentation characteristics including VFA, gas production, and degradability of nutrients. Results of this study can be used to acquire the optimum level of L-Gln for future *in-vivo* studies when maximum cell growth is targeted and to avoid possible toxic level(s).

## MATERIALS AND METHODS

### Experiment 1

#### Animals, diets, and ruminal liquor substrate

All animal procedures were approved by the Institution of Animal Care and Use Committee at Konkuk University (approval no. KU19075). Rumen liquor was obtained from three non-lactating Holstein cows at 30-month old (body weight [BW] = 735±25 kg) fed diets (Table 1) made of forage (F) and concentrate (C) on the basis of total mixed ration (TMR) at a F:C = 6:4 ratio *ad libitum* (exp. 1). These cows had free access to clean drinking water. Feed was provided daily at 09:00. The animals were surgically cannulated. Thus, rumen liquor could be obtained directly from the rumen. Two hours before feeding, ruminal contents from different cattle were combined and squeezed through two-layers of cheesecloth to remove feed particles. After filteration, the rumen liquor was transported to the laboratory using the pre-warmed (39°C) bottles that were vacuumed for making oxygen-free environment by inclusion of free carbon dioxide (CO_2_) into the headspace prior to the handling. McDougall’s [19] buffer solution (Table 2) was prepared. The pH of the buffer was adjusted to 6.8 under continuous flushing of CO_2_ for making anaerobic condition with constant magnetic stirring at 39°C. The buffer solution and rumen liquor were mixed at a ratio of 4:1 and 50 mL of the rumen inoculum was filled into pre-warmed (39°C) serum bottles. The headspace of the bottle was saturated with CO_2_. Bottles were capped with butyl rubber stoppers, sealed with center tear seals, and vortexed. Thereafter, samples were incubated in a thermostatically controlled incubator (39°C, 100 rpm) for 48 h. There was no feed contained. Only L-Gln (Daesang Co., Seoul, Korea) was incubated at two levels of 0 and 50 mM in three independent runs. Each level of 0 and 50 mM L-Gln was incubated in triplicate to improve the reliability of the assessment. The sampling time was 0, 6, 12, 24, 36, and 48 h, resulting in a total of 30 bottles per incubation (2 level×5 sampling time×3 replicates).

#### Ammonia-nitrogen analysis

Concentrations of ammonia-nitrogen (ammonia-N) (mg/100 mL) were determined according to protocol of Chaney and Marbach [20] in triplicates. For this, 0.01 mL of HgCl_2_ was added into 1 mL of rumen liquor sample. The mixture was then centrifuged at 3,000 rpm for 20 min at 4°C to collect the supernatant. After collection, 0.02 mL of the supernatant and ammonia N Standard solution (25, 50, 100, 200, and 400 ppm), 0.01 mL of alkali-hypochlorite, and phenol color reagent were added to a well of a plate and incubated at 50°C for 7 min. Thereafter, plates were read at 630 nm using a spectrophotometer (680, BIO-RAD, Hercules, CA, USA) to determine ammonia-N concentration. The degradation rate was calculated with the following formula: degradation rate = (36 h ammonia-N − 0 h ammonia-N)/(χ h ammonia-N − 0 h ammonia-N)×100.

### Experiment 2

#### Animals, diets, and ruminal liquor substrate

Rumen liquor from two non-lactating Korean native cows at 24-month old (BW = 475±25 kg) were exploited in exp. 2. Animals were fed diet (Table 3) of TMR *ad libitum*. Feed was provided daily at 09:00. All other conditions were the same as in exp. 1.

Two experiments were proceeded for determining the effects of L-Gln supplementation at levels of 0%, 0.5%, 1%, 2%, and 3% on fermentation characteristics, gas production, and rumen nutrient degradability. First, TMR was ground and 0.5 g was added to a bottle and incubated with L-Gln (Daesang Co., Korea) at 5 levels (0%, 0.5%, 1%, 2%, and 3%) in three independent runs. Each level was incubated in triplicate, whereas sampling time was 0, 6, 12, 24, 36, and 48 h, resulting in a total of 75 bottles per incubation (5 levels×5 sampling times×3 replicates). Second, TMR was ground, and 1.5 g was added to an ANKOM filter bag (filter bag 57, ANKOM, Macedon, NY, USA). Three independent bags were put in one bottle and incubated with L-Gln at five levels (0%, 0.5%, 1%, 2%, and 3%). Sampling time was 0, 12, 24, and 48 h, resulting in a total of 20 bottles per incubation (5 levels×4 sampling times). The method was modified with DAISY incubator (ANKOM, 2017).

#### Ammonia-N, pH, and volatile fatty acids analyses

Ammonia-N was measured according to the same procedure as explained in exp. 1. The pH was measured with a pH meter (MP230, Mettler Toledo, Columbus, OH, USA) for each sampling time (0, 6, 12, 24, 36, and 48 h). Volatile fatty acid concentration was determined according to method of Erwin et al [21] in triplicates. For this, 0.1 mL of phosphoric acid (25%, w/v) and 0.2 mL pivalic acid solution (2%, w/v) were added into 1 mL of rumen liquor sample. The mixture was then centrifuged at 12,000 rpm for 30 min at 4°C. The supernatant was collected and stored at −80°C before analysis. The VFA standard was prepared by adding each reagent (Supplementary Table 2-1). Thereafter, samples were analyzed to measure VFAs using a gas chromatograph (HP6890, Agilent, Mundelein, IL, USA).

#### Degradability of dry matter, crude protein, neutral detergent fiber, and acid detergent fiber analyses

Dry matter (DM) was determined followed by weighing a sample after drying at 60°C for 48 h in a dry oven. The *in-vitro* DM degradation calculation model was as follows: DM (%) = dried residue/sample weight × 100. Crude protein (CP) was determined using an elemental analyzer (EA 1110, CE instruments, USA). The *in-vitro* CP degradation was calculated with the following formula: CP (%) = N (%)×6.25. In detail, 3 mg of feed was weight with an ultrafine scale and added into a tin solid capsule (PN 240 06400, Thermo Scientific, Coatesville, PA, USA). Sulfanilammide standard (Supplementary Tables 2-4) was prepared the same as a sample for analysis. Neutral detergent fiber (NDF) and acid detergent fiber (ADF) were measured according to the procedure provided by ANKOM Technology. Doing these fiber analyses, 0.45 g of feed was added to ANKOM filter bag (filter bag 58, ANKOM, USA) followed by sealing the bags. Afterwards, the sealed bags were drowned in acetone for 10 min and then dried for another 10 min. After drying, samples were placed into the ANKOM fiber analyzer machine (ANKOM 200, ANKOM, USA). The NDF solution (Supplementary Table 2-2), 4 mL α-amylase, and 20 g sodium sulfate anhydrous were then added to the solution. The mixture was then heat activated at 100°C for 75 min. After heating, samples were washed in the machine for 12 min with 2 L of distilled water at 70°C (4 times of washing, 4 mL of α-amylase was added in the previous 2 times). Thereafter, each sample was drowned in acetone for 10 min, dried for another 10 min, and finally dried in a dry oven at 60°C for 48 h before weighing. The obtained samples were put into the analyzer after determining NDF and ADF (Supplementary Tables 2-3). The analyzer machine was set with the above method (ANKOM, 2017). In short, washing was conducted twice without α-amylase. Drowning in acetone was processed for 1 min, followed by drying for 10 min. Samples were weighted after drying in a drying oven at 60°C for 48 h. The *in-vitro* NDF (IVNDFD) and ADF (IVADFD) degradation were calculated with the following formula: NDF/ADF (%) = (dried residue − [filter bag weight × C])/sample weight×100. Correction (C) = dried blank bag weight/original blank bag weight. All the above samples were run in triplicate to increase the reliability of the assessment.

### Statistical analysis

Data of exp. 1 are reported as samples least-squares means± standard deviation. Data were analyzed using the MIXED procedure of SAS program (version 9.1; SAS institute Inc., Cary, NC, USA) as a randomized completely block design. The model was:


Yi(t)+μ+Ti+Ei(t)

where μ was average value, Ti was treatment value, and Ei(t) was error value. The fixed effect was Gln level. Polynomial orthogonal contrasts were used to determine the fermentation effect using contrast (estimate) option. The value indicated least squares means. The differences between means were determined using Tukey post hoc test. Statistical difference was accepted at p-value less than 0.05.

## RESULTS AND DISCUSSION

### Experiment 1

**Figure 1:**
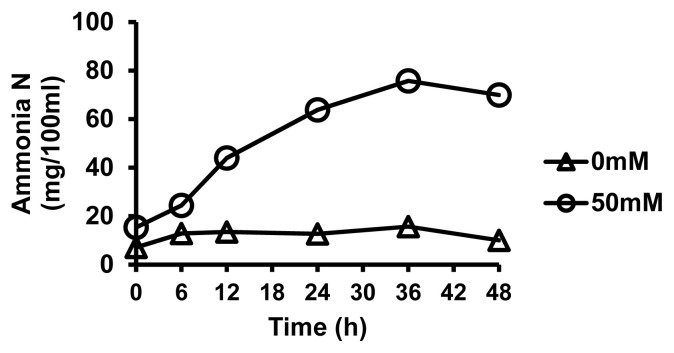
Ammonia N concentrations after *in-vitro* fermentation by ruminal bacteria in the group supplemented with 50 mM L-glutamine. Effects of incubation time were observed (p<0.001) (exp. 1).

Ammonia-N concentrations were increased (p<0.001) over time when Gln was supplemented at 0.5% (Figure 1), indicating constant increase in Gln degradation by increasing incubation time from 0 to 48 h. Ammonia-N concentration was increased between 6 and 24 h, showing a peak at 36 h. At 36 h, ammonia-N concentration was 75.80 mg/100 mL greater in bottles containing Gln than those without Gln. A total of 100 mg/100 mL of ammonia-N was expected from complete degradation of 50 mM Gln. Similar phenomenon in cattle supplemented with urea-molasses-mineral block was observed [22]. The mechanism behind increased ammonia-N concentration over time could be attributed to deamination of the substrate (L-Gln) by rumen liquor microbes. Also, as expected, a steady concentration of ammonia-N at 48 h after a peak at 36 h could be explained by a response to the balance between deamination and the utilization of ammonia-N by microbes, consistent with results of Wang et al [23] using limited AAs *in-vitro*. On the other hand, Gilbreath et al [24] stated that ruminal microbes of adult steers do not degrade extracellular L-citrulline and have a limited ability to metabolize extracellular L-Glu. The deamination occurs only during the short period of incubation.

The degradation rate by time was calculated based on the percentage of ammonia-N value at each incubation time when the maximum value was set at 36 h assuming 100% degradation. L-Gln degradation rate was 2% at 6 h, 21% at 12 h, 76% at 24 h, and 100% at 36 to 48 h. Given that the ruminal retention time was usually 12 to 24 h in case of powdery AAs, the present study showed degradation rates of approximately 21% to 76% (Table 4). Generally, degradation of AAs results in the amino group either being incorporated into other nitrogenous compounds or being excreted as ammonia or urea. Hence, an understanding of AA degradation provides insights on interrelationships between metabolic pathways and helps explain deficiencies in AA metabolism [25]. In this study, we observed a linear constant increase in degradation of L-Gln as time passed from 6 h to 36 h of incubation and then remained steady with a degradation rate of 100% at 36 to 48 h. The degradation rate of Gln in the culture medium was 76%, postulating the availability of two thirds of Gln by rumen microbes before digesta may be bypassed (in 24 h) from the rumen based of the obtained result. Thus, attention is required about supplementation of Gln to the diet in future *in-vivo* studies. The rest of degradation may occur in the intestine in an actual animal study.

### Experiment 2

This is the first study that tests several incubation times from 0 to 48 h with five levels of L-Gln supplementation (0%, 0.5%, 1%, 2%, and 3%), and the contrast of L-Gln including linear and quadratic effects at once. Thus, fluctuations in results would be inevitable. Given this, it might be difficult to set only one level of supplementation as a final result. Also, the fact that feed usually will maintain in the rumen for about 24 h, any recommendations should consider this average feed remaining time that can fluctuate by feed type and ingredients. Therefore, we provided a sectional conclusion based on each supplemental level for each trait before drawing an overall conclusion.

The pH range of the culture medium was reduced from 6.78 to 6.03 as the incubation time elapsed. There was a significant difference in the pH value of each treatment regardless of incubation time (p<0.05) (Table 5). The suitable range of pH value in rumen liquor is about 5.5 to 7.5 for microbial growth purpose. Any change in its pH can strongly affect the vigor of rumen microbes and their activity [23]. When the pH value is more than 7, utilization of ammonia is reduced [12]. Meanwhile, when the pH value is less than 6.2, the degradation activity of cellulose is decreased, causing slower degradation of cellulose wall, lower growth of microorganisms, and lower production of VFAs and methane [25,26]. In addition, microorganisms might be inactivated when the pH is 5 or lower. The non-protein nitrogen fraction of feed is rich in Gln and other components. Consequently, it is likely that the add-up effects of L-Gln supplementation in this study can facilitate buffering capacity in the rumen as reported by general feature of some other AAs effects [27]. Accordingly, in this study, using L-Gln supplementation in culture medium could maintain pH within optimal values. Results of this study suggest that changes in pH depending on treatment were significantly different. However, all samples maintained pH values within its normal range, suggesting that L-Gln might not affect rumen fermentation in future *in-vivo* study. However, this result should be interpreted with caution before performing any actual *in-vivo* study due to effects of other feed ingredients on pH values. Although the pH range in this study was fairly consistent with the study of Zhang et al [18] that reported effects of branched chain AAs supplementation including valine, leucine, and isoleucine on *in-vitro* ruminal fermentation, their study showed unchanged pH as provision amount was increased from 0 to 2, 4, 7, and 10 mmol/L. From a pH perspective, no restriction in L-Gln supplementation up to 3% can be considered.

Generally, total gas production per incubation time tended to decrease in all groups compared to the control groups except for the 3% Gln group at 36 h. On the other hand, total gas production tended to increase in all treatment groups with increasing incubation time. In particular, from 36 h to 48 h of incubation, Gln treated group showed a decrease (p<0.01) of total gas production compared to the control group while diet, linear, and quadratic effects were all significant. Higher (p<0.001) total gas in the 0.5% Gln group at 3 and 6 h compared to the control revealed that at early incubation time, 0.5% L-Gln could cause an increase in total gas production which was supported by observing higher total VFA production at the same time of incubation (Table 6). When treatment groups were compared, the 3% Gln level group showed the lowest gas production at 48 h (Table 5). Generally, total gas production, VFA production, and degradability are positively correlated with each other. Higher *in-vitro* total gas production reveals higher fermentation end products that could be preferable [11]. Total gas results can also be estimated from molar percentage of VFA [12,28]. Protein and starch are digested by microorganisms such as bacteria, protozoa, and fungi to produce sugar and AAs. Sugar and AAs sourced from diet or by microbial metabolism are digested by microorganisms again and then fermented VFA, H_2_, and CO_2_. In this study, the inhibitory effect of L-Gln supplementation at a high dosage (2% to 3%, Table 5) to the culture medium on total gas production can be explained by a decrease of hydrogen supply for methanogens [12,29]. Janssen [30] explained the influence of hydrogen on rumen CH_4_ formation and fermentation balances through microbial growth kinetics and fermentation thermodynamics. Janssen [30] stated that hydrogen gas produced during microbial fermentation of feed is used as an energy source by methanogenic archaea (i.e., methanogens), which produce CH_4_. Therefore, in this study, Gln was competing with methane to use hydrogen in light of increase in propionic acid (Table 6) as supplementation rate of Gln increased. Given the above discussion and as CH_4_ is one component of total gas produced, CH_4_ production might be decreased by Gln supplementation. Most of the supplemented Gln might be transformed to Glu via GS-GOGAT pathway which is a dominant pathway for the ammonium assimilation in rumen bacterial ecology [31]. According to the pathway, when Gln is transformed to Glu, one molecule of nicotinamide adenine dinucleotide phosphate (NADPH) is used [31]. Various Glu fermentation pathways were reported, and a few pathways consume H^+^ which is also the substrate for the methanogenesis [32,33]. As such, the Gln supplementation can reduce total gas production (TGP) not only because of the NADPH consumption during the GS-GOGAT pathway, but also the competition between Gln (or Glu) utilizing bacteria (e.g., *Clostrkiium aminophilum*, which is one of the most ammonia-producing rumen bacteria prefer Glu and Gln as a carbon source) and methanogens for hydrogen [30,32,33]. Therefore, Gln supplementation can promote protein synthesis and propionate production (Table 6) and decrease CH_4_ formation as propionate can compete with CH_4_ for hydrogen [28,30,31]. Furthermore, protozoa can affect activities of some methanogens [12], contributing about 10 to 25% of ruminal CH_4_ production [34]. However, the reason why L-Gln could not significantly alleviate total gas in mid incubation time of 12 and 24 h to the authors remained unclear. One hypothesis is that the ruminal fermentation process is inefficient because it produces some final products such as methane gas [35] and excess ammonia [36]. Consistently with the present results, Megías et al [37] have examined *in-vitro* gas production by supplementing by-products and indicated that highly N-enriched substrates produce less gas than substrates with lower N. This was also confirmed in the present study. Depending on such hypothesis, if higher gas production is targeted, due to increasing incubation time, control and up to 1% L-Gln supplementation can be considered for future *in-vivo* research. Vice versa, if mitigation of gas production is targeted, higher levels of L-Gln up to 3% is preferable. Therefore, setting an optimal level of L-Gln supplementation in order to affect total gas production depends on the aim of each study. In the present *in-vitro* study, since higher total gas production is associated with higher VFA production, control and 0.5% L-Gln supplementations in early incubation time can be studied in future research. Collectively, as the level of Gln increases, it has a negative (reduced) effect on total gas production. This should be considered in further *in-vivo* study.

Ammonia-N tended to increase in control and all treatment groups with increasing incubation time. The concentration of ammonia-N increased (p<0.05) with increasing L-Gln level at 48 h of incubation, showing both linear and quadratic effects (Table 5). Mainly, the highest ammonia-N concentrations were observed in groups with 2% and 3% L-Gln supplementations. Ammonia-N concentration is also considered as a colligation indicator of degradation and utilization of nitrogen source by rumen microbes [23]. When the activity of microorganisms in the rumen is increased, ammonia-N is produced as CP and AA in the feed are decomposed. Degraded ammonia-N is used for microbial protein formation [11]. Crude protein content in feed is known to affect ammonia-N concentration [38]. The optimal ammonia-N concentration for microbial protein synthesis is somewhat different, ranging from 5 mg/100 mL to 29 mg/100 mL. In addition, when ammonia-N concentration was greater than 84 mg/100 mL, the capacity of liver reached its limit in the treatment group and poisoning symptoms appeared within 30 minutes after feeding [39]. In our study, concentrations of ammonia-N in all treatment group were lower than its toxic level. However, 2% and 3% Gln groups had the highest concentrations of ammonia-N while the 0.5% Gln group had lower concentration of ammonia-N than the control group. The results of exp. 2 are consistent with the results of exp. 1, indicating that ammonia-N concentration was increased with increasing incubation time. However, in an actual *in-vivo* study, higher concentrations of ammonia-N should be avoided due to possible toxicity effects. Thus, 2% and 3% L-Gln might need to be avoided. Higher concentration of ammonia-N in 2% and 3% groups may also imply the inability of microorganisms to use Gln when the higher synthesis of protein is targeted. This phenomenon could be due to an unequal availability of energy and ammonia, making them less useful in the process of microbial protein synthesis as suggested by Syamsi et al [11] after supplementing various meal protein sources with different protein-energy synchronization index in dairy ration. In the present study, the low and high values of ammonia-N concentration fell within its suitable range in culture medium in both experiments (Figure 1; Table 5).

In the present study, acetate, propionate, and total VFA tended to increase with elevated Gln level and incubation time, particularly from 12 to 48 h (p<0.001) (Tables 6, 7). Principally, acetate showed quadratic effects with maximum amounts in control, 0.5%, 2%, and 3% L-Gln supplementation whereas propionate showed higher amounts mainly in 0.5% and 1% L-Gln groups. This increase is in line with the result of exp. 1 showing an increased degradation rate of L-Gln over time. Thus, degradation of L-Gln could provide a good substrate for VFA production. Microorganisms are known to grow and develop by utilizing feed substrates and producing VFAs. Improving the performance of rumen microorganisms will be in line with increasing rumen fermentation products, ultimately increasing the productivity of the animals [11]. VFAs are the end products of ruminal microbial fermentation. They are used as major sources of ruminant metabolism energy [27]. Main VFAs that are targeted to increase include acetate and propionate rather than branched chain VFAs that are present in total VFA in very small amounts. Acetate is used for fat synthesis. Propionate is known to be used for gluconeogenesis. Gln is temperature sensitive. It can be transformed into Glu within the rumen and used as a precursor for Glu and alanine in the skeletal muscle. Gln and alanine pathways produce acetate and propionate [39]. Taken together, these results indicate that increasing concentration of L-Gln can increase VFA production. Obtained results are consistent with results of Zain et al [40] *in-vivo* on sheep and Zhang et al [14] *in-vitro*, reporting increased acetate, propionate, and total of branched chain VFA by supplementing branched chain AAs. The reason for this increase may be due to expedition of L-Gln substrate degradation. Syamsi et al [11] have stated the role of cellulolytic bacteria in inducing the production of VFAs, with acetic acid as a corresponding VFA to digest structural carbohydrate. Another reason for higher acetate concentration in the 0.5% Gln group could be attributed to the higher fiber degradability including NDF and ADF in the same group. These results suggest that L-Gln should be supplemented to ruminants at 0.5% in further *in-vivo* studies. In this study, total VFAs and individual VFAs yielded more quadratic increases, particularly by increasing incubation time from 24 h to 48 h as supplementation amount of L-Gln increased. The lowest amount of VFAs was produced when L-Gln supplementation was set at 1% level. This phenomenon could be explained by congestion of VFAs by increasing supplementation of L-Gln from 0.5% to 1%. However, the quadratic increase when Gln supplementation was set at 2% and 3% levels remained unclear. One hypothesis could be that branched chain VFAs including isobutyric, isovaleric, and 2-methylbutyric acid are produced due to forage fiber degradation by microorganisms and degradation of branched chain AAs including valine, leucine, and isoleucine [17,18] in the rumen primarily originating from dietary true protein degradation [18] (herein, L-Gln). Furthermore, branched chain VFA is a result of AA deamination in the rumen. Therefore, an increase in branched chain VFA level in the rumen can be induced by supplementing high protein source in the ration [41], herein L-Gln. Isobutyrate and butyrate followed similar pattern of alterations in culture medium (Table 6). Comparable significant values where at least diet effect was accompanied by either linear or quadratic effects except for isobutyrate at 12 h were observed from 3 h to 48 h, whereas 1% L-Gln supplementation showed the lowest values of butyrate and isobutyrate. Isovalerate and valerate concentrations showed exactly the same pattern of alterations as butyrate and isobutyrate, respectively. Given positive effects of L-Gln after different incubation time on acetate at 0.5 and 2% to 3% levels of Gln and propionate at both 0.5% and 1% levels of Gln *in-vitro*, recommendation of the optimum level of Gln supplementation should be obtained with caution in order to obtain desired VFAs prior to actual *in-vivo* studies. However, most of significant higher values in each VFA were observed in the 0.5% L-Gln supplementation group. With respect to the concentrations of other branched chain VFAs, the optimum level of Gln supplementation could be set at both 0.5% and 3% considering results of the present study. Since lower amount of any supplemented product is desirable economically, provision of 0.5% L-Gln could be the best choice. However, this level should be confirmed in further *in-vivo* studies.

Degradability results are provided in Table 7. Control and 0.5% group after all incubation time, 1% group after up to 24 h incubation, and 2% group after 12 h incubation showed the highest degradability of DM with a quadratic effect (p< 0.001). NDF degradability showed fluctuations from time to time, whereas it achieved the maximum values when L-Gln was added at 2% after 12 h incubation, 0.5% and 1% after 24 h incubation, and 0.5% and control after 48 h of incubation, showing a quadratic effect (p = 0.005). Similar pattern was obtained in ADF degradability while 0.5% and 3% L-Gln supplementation after 48 h of incubation showed quadratic effect (p = 0.003). L-Gln also increased CP degradability (p<0.001), with the highest degradability found in the 3% L-Gln group regardless of incubation time (p<0.05). Degradability of DM rely on the rate of AA supplementation. In this study, DM degradability was declined when the level of added L-Gln exceeded 2%. Using branched chain AAs supplementation *in-vitro*, consistent decline in DM digestibility was observed when the level of supplementation exceeded 2 mmol/L [18]. Although 1% Gln group after 12 and 24 h of incubation and 2% Gln group after 12 h of incubation showed similar DM degradability as the 0.5% Gln group, the 0.5% Gln group showed higher DM degradability regardless of incubation time, revealing a constant influence of L-Gln supplementation at the corresponding level. Higher DM degradability after all incubation time were observed in the 0.5% Gln group, indicating that the optimal concentration of L-Gln for digestion by ruminal microorganisms should be approximately 0.5%. Yang et al [42] have reported the paucity of information regarding the effects of adding branched chain AAs on ruminal fermentation characteristics both *in-vitro* and *in-vivo*. In the present study, although fluctuations in NDF degradability were observed among incubation time, higher degradability of NDF was found in the 0.5% Gln group after 24 to 48 h incubation, the 1%, Gln group after 24 h incubation, and the 2% Gln group after 12 h incubation, indicating that ruminal microorganisms in culture medium could benefit from direct supplementation of L-Gln when the supplementation level was decreased from 2% to 0.5% and when the incubation time was increased. Consistent with our results for low level of L-Gln supplementation (0.5%), Zhang et al [18] have reported higher degradability of NDF when valine and isoleucine are supplemented at low concentration of 2 mmol/L compared to 4, 7, and 10 mmol/L levels. This was also reported by Chen et al [43]. Similar pattern in increasing NDF degradability by ascending incubation time and descending L-Gln level was also observed in ADF degradability. The reason behind a sudden quadratic increase in ADF degradability in the 3% group at 48 h remains uncertain. However, decreased NDF and ADF degradabilities in 1% and 2% groups at 48 h after observing their increases in the group with 0.5% Gln supplementation might be explained by saturation of culture medium between the ratio of supplementation and activity of microorganisms for fiber fraction degradation. These results revealed a balance between supplementation rate and microorganism activity. Thus, 0.5% L-Gln supplementation is suitable when fiber degradability is targeted. Consistently, Yang et al [42] have reported similar effects of direct provision of breached chain AAs on degradability of fiber fraction *in-vitro*. With regard to CP degradability, *Ruminobacter amylophilus*, *Prevotella ruminicola*, *Butyrivibrio fibrisolven*, *Strptococcus bovis*, and *Peptostreptococcus* are typical feed protein degradation bacteria that can grow using AAs and peptides. These bacteria account for more than 10% of digestive bacteria [42]. The growth of microorganisms is influenced by energy, ammonia, and cofactors. Energy and ammonia are main factors that are mutually limiting. Thus, means that an unequal availability of energy and ammonia would keep them less useful in the process of microbial protein synthesis [11]. Symasi et al [11] indicated that both ammonia and energy have to be available simultaneously (synchronous) to achieve maximum microbial protein production. Synchronized availability of energy and ammonia is influenced by the rate of degradation of protein (herein L-Gln) and carbohydrate degradation products as energy sources (herein VFAs). Hence, the reason behind higher degradability of CP in the 3% L-Gln group can be attributed to the synchronization of L-Gln with substrate for maximizing bacteria growth. On the other hand, branched chain VFAs and total VFA can be used to estimate the degree of protein degradation [17] as shown in the present study. Another hypothesis that may explain higher CP degradability could be higher VFAs and ammonia-N in the corresponding group (3% L-Gln). Furthermore, higher ammonia-N is associated with lower microbial proteins due to saturation of microorganisms’ inability of using excess N to produce microbial proteins as stated by Symasi et al [11]. Considering our degradability results, 0.5% L-Gln supplementation could be beneficial to improve DM, CP, ADF, and NDF degradability, although this should be confirmed in further *in-vivo* studies.

One limitation of this study was inconsistency in some results including VFA and gas production results. The inconsistent results might be resulted from the ice treatment during the *in-vitro* procedure. When the incubation bottles were taken from the shaking incubator, the bottles were put on the ice to stop the further microbial fermentation. But, the TGP measurements were conducted when the bottles were on the ice. Accordingly, the TGP decreased according to the decreased temperature of the headspace. Another limitation was regarding microbial activity analysis, which have not performed in this study. The reason for this was due to unavailability of previous study having several various levels of L-Gln supplementation with very wide range of incubation times. As such, microbial activity was not the first important criteria to be measured while no information was available with respect to the total gas production, L-Gln degradation, ammonia production, and VFA production etc. Thus, microbial activity should be further examined in future study after taking into account of the results of this study.

## CONCLUSION

No toxicity was observed based on determined values of pH and ammonia-N (NH3) in all treatment groups after supplementation of L-Gln at high levels of 2% and 3% *in-vitro*. However, 2% and 3% L-Gln supplementation increased ammonia-N numerically which could potentially cause toxic effects in an actual *in-vivo* study. Thus, before making an overall decision of using high levels of supplementation, caution should be given over supplementation of 2% to 3% L-Gln due to other influencing cofactors in the rumen in a further *in-vivo* study. Overall, the supplementation of L-Gln at 0.5% is recommended due to its positive effects on overall traits assessed including rumen fermentation end products (VFAs) and degradability of nutrients. Thus, 0.5% Gln should be tested in further *in-vivo* studies.

## Figures and Tables

**Table 1:** Chemical compositions of the experimental diet (exp. 1)

Chemical composition	Forage (F)	Concentrate (C)	Total mixed ration (F:C = 6:4)
Dry matter	88.1	86.6	62.4
Crude protein	4.1	13.5	10.0
Crude fat	0.7	3.7	2.6
Neutral detergent fiber	31.2	8.8	16.9
Acid detergent fiber	4.4	6.0	5.0

The values are in %.

**Table 2:** Chemical compositions of McDougall’s buffer (exp. 1)

Ingredient composition	Amount (g/L DH_2_O)
NaHCO_3_ (Sodium carbonate)	9.80
Na_2_HPO_4_ (Sodium phosphate)	3.69
KCl (Potassium chloride)	0.57
NaCl (Sodium chloride)	0.47
MgSO_4_ (Magnesium sulfate)	0.12

**Table 3:** Ingredients and chemical compositions of the diet (exp. 2)

Item	Amount
Ingredients (% dry matter)	
Corn flake	20.6
Soybean curd residue	18.3
Yeast	18.3
Rice straw	11.4
Soybean pellet	6.9
Soybean hulls	6.9
Corn gluten meal	6.9
Rice flour	4.5
Bakery by-product	2.3
Sesame oil meal	2.3
Limestone	1.0
NaCl	0.6
Chemical composition (%)	
Moisture	38.1
Crude protein	14.2
Neutral detergent fiber	41.7
Acid detergent fiber	23.3
Crude fat	2.7
Crude ash	5.8
Glutamic acid	1.3

**Table 4:** Calculated degradation rate of L-glutamine (exp. 1)

Time (h)	Degradation rate (%)
6	2
12	21
24	76
36	100
48	100

Degradation rate = (36 h ammonia N − 0 h ammonia-N)/(χ h ammonia-N − 0 h ammonia-N) × 100.

**Table 5:** Effects of L-glutamine supplementation levels on pH, total gas, and ammonia-N concentration

Items	Time (h)^1)^	Treatments (%)^2)^					SEM	p-value		
	
		Control	0.5	1	2	3		Diet	Linear	Quadratic
pH	0	6.74^b^	6.78^a^	6.68^c^	6.69^c^	6.69^c^	0.006	<0.001	<0.001	<0.001
	3	6.73^a^	6.70^ab^	6.67^c^	6.70^b^	6.67^c^	0.005	<0.001	<0.001	0.014
	6	6.53^a^	6.51^ab^	6.47^c^	6.52^a^	6.49^bc^	0.006	<0.001	0.003	0.012
	12	6.28^a^	6.30^a^	6.20^b^	6.22^b^	6.23^b^	0.007	<0.001	<0.001	<0.001
	24	6.15^a^	6.16^a^	6.06^b^	6.07^b^	6.08^b^	0.005	<0.001	<0.001	<0.001
	36	6.14^a^	6.16^a^	6.05^c^	6.09^b^	6.07^bc^	0.006	<0.001	<0.001	<0.001
	48	6.13^b^	6.17^a^	6.03^d^	6.06^c^	6.04^cd^	0.006	<0.001	<0.001	<0.001
Total gas (mL)	3	0.17^b^	2.33^a^	1.17^ab^	1.00^ab^	0.67^b^	0.291	0.004	0.907	0.704
	6	12.83^b^	15.33^a^	12.58^bc^	11.08^c^	12.25^bc^	0.367	<0.001	0.421	0.828
	12	23.25	23.58	23.33	22.83	23.50	0.242	0.284	0.975	0.904
	24	38.33	38.67	37.00	35.33	38.67	1.578	0.533	0.785	0.301
	36	64.67^a^	49.33^ab^	48.67^ab^	43.33^b^	53.67^ab^	3.921	0.018	<0.001	<0.001
	48	77.33^a^	68.67^ab^	56.67^bc^	48.00^c^	47.33^c^	3.664	<0.001	<0.001	<0.001
Ammonia-N (mg/100 mL)	0	5.73^c^	7.01^b^	7.92^b^	9.30^a^	8.86^ab^	0.302	0.004	0.233	0.38
	3	7.32^b^	7.59^b^	7.36^b^	7.89^ab^	8.23^a^	0.166	0.035	0.684	0.873
	6	5.30^c^	3.93^d^	5.90^ab^	6.21^a^	6.44^ab^	0.299	<0.001	0.161	0.352
	12	3.75^d^	4.18^cd^	4.19^c^	5.40^a^	6.33^a^	0.090	<0.001	0.04	0.705
	24	7.76^c^	7.83^c^	8.28^b^	9.93^a^	11.43^a^	0.130	<0.001	0.015	0.635
	36	14.64	13.48	14.29	14.73	16.37	0.872	0.631	0.548	0.598
	48	19.98	19.79	19.66	22.27	22.83	1.597	0.472	0.003	0.002

The value indicate least squares means (n = 3).

SEM, standard error of the mean.

1) Time, incubation time.

2) Treatments: control (0%), 0.5%, 1%, 2%, and 3% L-glutamine dry matter basis.

a–d The values with different superscripts in the same row differ significantly (p<0.05).

**Table 6:** Effects of L-glutamine supplementation levels on acetate, propionate, isobutyrate, butyrate, isovalerate, valerate, and total volatile fatty acids concentrations

Items	Time (h)^1)^	Treatments (%)^2)^					SEM	p-value		
	
		Control	0.5	1	2	3		Diet	Linear	Quadratic
Acetate (mM)	0	18.80	18.90	18.68	18.98	18.86	0.070	0.651	0.904	0.984
	3	26.57^b^	29.79^a^	25.26^b^	26.44^b^	27.26^b^	0.286	0.003	0.471	0.253
	6	38.14^b^	41.80^a^	38.26^b^	37.32^b^	38.34^b^	0.274	0.002	0.041	0.841
	12	53.63^b^	55.26^b^	55.38^b^	56.81^ab^	59.16^a^	0.378	0.005	<0.001	0.724
	24	70.24^bc^	70.41^bc^	68.04^c^	71.30^b^	74.43^a^	0.332	<0.001	<0.001	<0.001
	36	76.8^a^	76.08^ab^	72.55^c^	76.00^ab^	76.93^a^	0.448	0.036	0.328	<0.001
	48	76.79^bc^	77.17^ab^	75.46^c^	78.63^ab^	80.77^a^	0.284	<0.001	<0.001	0.012
Propionate (mM)	0	3.45	3.46	3.40	3.44	3.43	0.027	0.652	0.906	0.984
	3	6.55^b^	7.51^a^	6.31^b^	6.50^b^	6.71^b^	0.110	0.003	0.473	0.253
	6	10.88^c^	11.74^a^	11.42^b^	10.67^c^	10.55^c^	0.106	0.002	0.042	0.841
	12	15.08^c^	15.38^c^	16.81^a^	16.29^b^	16.17^b^	0.146	0.005	<0.001	0.723
	24	18.97^c^	18.94^c^	19.65^a^	18.96^c^	19.58^ab^	0.128	<0.001	<0.001	<0.001
	36	20.34^a^	20.20^ab^	20.23^ab^	19.94^b^	20.05^ab^	0.172	0.036	0.328	<0.001
	48	20.17^bc^	20.15^bc^	20.72^a^	20.40^ab^	20.63^a^	0.109	<0.001	<0.001	0.012
Isobutyrate (mM)	0	0.08	0.08	0.08	0.08	0.08	0.002	0.321	0.752	0.991
	3	0.11^b^	0.12^a^	0.10^c^	0.10^bc^	0.11^b^	0.003	<0.001	0.200	0.020
	6	0.14^a^	0.14^a^	0.13^b^	0.13^b^	0.13^b^	0.002	<0.001	0.003	0.103
	12	0.18	0.18	0.17	0.18	0.18	0.005	0.194	0.442	0.028
	24	0.30^a^	0.30^a^	0.26^b^	0.28^a^	0.30^a^	0.005	<0.001	0.905	<0.001
	36	0.39^a^	0.39^ab^	0.34^d^	0.37^bc^	0.36^cd^	0.007	<0.001	<0.001	<0.001
	48	0.43^a^	0.43^a^	0.39^b^	0.41^a^	0.42^a^	0.005	<0.001	0.174	<0.001
Butyrate(mM)	0	1.54	1.55	1.50	1.54	1.53	0.010	0.063	0.989	0.836
	3	2.31^b^	2.63^a^	2.04^c^	2.27^bc^	2.46^ab^	0.049	<0.001	0.780	0.014
	6	3.58^abc^	3.84^a^	3.35^c^	3.46^bc^	3.68^ab^	0.052	0.001	0.767	0.014
	12	5.79^c^	5.70^c^	5.82^c^	6.34^b^	6.78^a^	0.083	<0.001	<0.001	0.005
	24	7.64^bc^	7.35^cd^	7.11^d^	7.84^b^	8.36^a^	0.064	<0.001	<0.001	<0.001
	36	8.20^a^	7.98^ab^	7.56^b^	8.29^a^	8.47^a^	0.107	0.003	<0.001	<0.001
	48	8.08^c^	7.93^c^	7.83^c^	8.49^b^	8.87^a^	0.073	<0.001	<0.001	<0.001
Isovalerate (mM)	0	0.10	0.10	0.14	0.10	0.10	0.018	0.130	0.434	0.010
	3	0.15^b^	0.18^a^	0.13^c^	0.15^bc^	0.16^b^	0.007	<0.001	0.448	0.053
	6	0.21^a^	0.21^a^	0.19^b^	0.20^b^	0.20^b^	0.004	<0.001	0.067	0.093
	12	0.28	0.27	0.26	0.27	0.27	0.009	0.140	0.623	0.123
	24	0.47^a^	0.46^ab^	0.41^c^	0.45^b^	0.47^ab^	0.008	<0.001	0.761	<0.001
	36	0.65^a^	0.63^ab^	0.55^d^	0.60^bc^	0.58^cd^	0.014	<0.001	<0.001	<0.001
	48	0.71^a^	0.71^a^	0.63^b^	0.68^a^	0.70^a^	0.011	<0.001	<0.001	0.007
Valerate (mM)	0	0.10	0.10	0.10	0.10	0.10	0.002	0.384	0.794	0.936
	3	0.17^ab^	0.19^a^	0.15^c^	0.16^bc^	0.18^ab^	0.007	<0.001	0.784	0.003
	6	0.31^ab^	0.32^a^	0.28^c^	0.29^bc^	0.29^bc^	0.009	0.002	0.002	0.021
	12	0.41	0.41	0.42	0.43	0.43	0.012	0.168	0.002	0.501
	24	0.53^ab^	0.53^ab^	0.51^c^	0.52^bc^	0.55^a^	0.009	0.002	0.012	<0.001
	36	0.61^a^	0.60^a^	0.56^b^	0.59^ab^	0.58^ab^	0.013	0.004	0.007	<0.001
	48	0.62^a^	0.62^a^	0.58^b^	0.62^a^	0.64^a^	0.011	0.002	0.002	<0.001
Total VFA (mM)	0	24.07	24.19	23.89	24.24	24.11	0.110	0.790	0.928	0.963
	3	35.86^b^	40.41^a^	33.99^b^	35.62^b^	36.87^ab^	0.461	0.002	0.553	0.156
	6	53.26^b^	58.05^a^	53.64^b^	52.07^b^	53.19^b^	0.440	0.002	0.059	0.55
	12	75.38^b^	77.20^b^	78.85^b^	80.33^ab^	82.99^a^	0.626	0.003	<0.001	0.473
	24	98.15^bc^	97.99^bc^	95.98^c^	99.34^b^	103.7^a^	0.531	<0.001	<0.001	<0.001
	36	107.0^a^	105.9^ab^	101.8^b^	105.8^ab^	107.0^a^	0.743	0.021	0.226	<0.001
	48	106.8^bc^	107.0^bc^	105.6^c^	109.2^ab^	112.0^a^	0.477	<0.001	<0.001	0.003

The value indicate least squares means (n = 3).

SEM, standard error of the mean; VFA, volatile fatty acids.

1) Time, incubation time.

2) Treatments: control (0%), 0.5%, 1%, 2%, and 3% L-glutamine dry matter basis.

a–d The values with different superscripts in the same row are differ significantly (p<0.05).

**Table 7:** Effects of L-glutamine supplementation levels on DM, NDF, ADF, and CP degradability

Items	Time (h)^1)^	Treatments (%)^2)^					SEM	p-value		
	
		Control	0.5	1	2	3		Diet	Linear	Quadratic
DMD (%)	0	5.69^a^	4.02^a^	4.71^a^	4.74^a^	5.25^b^	0.989	0.002	0.949	0.566
	12	40.76^c^	45.58^ab^	46.04^a^	46.16^a^	40.88^c^	1.143	0.015	0.607	0.001
	24	58.50^d^	66.36^ab^	68.12^a^	63.52^b^	61.46^c^	1.199	0.003	0.831	<0.001
	48	69.93^bc^	75.44^a^	69.79^c^	65.64^cd^	70.21^bc^	1.696	0.033	0.120	0.323
NDFD (% DM)	0	11.03	10.03	11.75	9.67	9.52	0.294	0.121	0.601	0.648
	12	50.58	54.79	53.21	57.34	52.04	0.555	0.062	0.055	0.668
	24	70.65	77.65	80.11	75.1	73.38	1.035	0.339	0.846	0.102
	48	85.00^ab^	86.88^a^	79.11^c^	78.35^cd^	84.52^b^	1.577	0.025	0.682	0.005
ADFD (% DM)	0	10.59	9.99	11.79	10.10	12.65	0.223	0.697	0.207	0.222
	12	48.18^e^	52.28^bc^	50.95^d^	54.83^a^	52.63^b^	0.403	0.054	0.013	0.131
	24	68.85	75.01	77.08	72.66	72.63	0.726	0.759	0.580	0.918
	48	83.07^ab^	84.83^a^	76.49^c^	74.59^c^	85.44^a^	1.053	0.014	0.435	0.003
CPD (% DM)	0	7.68	1.79	0.68	0.00	16.39	0.223	0.458	0.016	0.03
	12	45.52^c^	51.94^b^	53.34^a^	42.22^e^	44.00^d^	0.095	0.037	0.900	0.481
	24	66.68^c^	77.73^a^	76.63^b^	68.63^c^	65.53^d^	0.083	0.007	0.836	0.925
	48	80.75^b^	87.46^a^	79.66^c^	68.81^e^	76.73^d^	0.063	<0.001	<0.001	0.922

The value indicate least squares means (n = 3).

DM, dry matter; NDF, neutral detergent fiber; ADF, acid detergent fiber; CP, crude protein; DMD, degradability of dry matter; NDFD, degradability of neutral detergent fiber; ADFD, degradability of acid detergent fiber; CPD, degradability of crude fiber.

1) Time, incubation time.

2) Treatments: control (0%), 0.5%, 1%, 2%, and 3% L-glutamine DM basis.

a–e The values with different superscripts in the same row are differ significantly (p<0.05).

## References

[1] Xiao D, Zeng L, Yao K, Kong X, Wu G, Yin Y (2016). The glutamine-alpha-ketoglutarate (AKG) metabolism and its nutritional implications. Amino Acids.

[2] Shah AM, Wang Z, Ma J (2020). Glutamine metabolism and its role in immunity, a comprehensive review. Animals.

[3] Antonio J, Street C (1999). Glutamine: A potentially useful supplement for athletes. Can J Appl Physiol.

[4] Kreider RB (1999). Dietary supplements and the promotion of muscle growth with resistance exercise. Sports Med.

[5] Akbarnezhad A, Ravasi AA, Aminian Razavi TD, Nourmohammadi I (2006). The effect of creatine and glutamine supplements on athletic performance in elite wrestlers after one acute period of weight losing. Harakat.

[6] Ramezani Ahmadi A, Rayyani E, Bahreini M, Mansoori A (2019). The effect of glutamine supplementation on athletic performance, body composition, and immune function: A systematic review and a meta-analysis of clinical trials. Clin Nutr.

[7] Wu G (2009). Amino acids: metabolism, functions, and nutrition. Amino Acids.

[8] Wu G, Bazer FW, Johnson GA (2011). Triennial Growth Symposium: important roles for L-glutamine in swine nutrition and production. J Anim Sci.

[9] Kim YS, Lee JS, Lee YS (2018). Effect of glutamine on heat-shock protein beta 1 (HSPB1) expression during myogenic differentiation in bovine embryonic fibroblast cells. J Food Sci Biotechnol.

[10] Lobley G, Hoskin S, McNeil CJ (2001). Glutamine in animal science and production. J Nutr.

[11] Syamsi AN, Waldi L, Widodo HS, Harwanto (2019). Branched chain volatile fatty acids profile of rumen fluids supplemented by different meal protein sources and protein-energy synchronization index. IOP Conference Series: Earth Environ Sci.

[12] Kim JY, Ghassemi Nejad J, Park JY (2018). In vivo evaluation of garlic (Allium sativum) supplementation to rice straw-based diet on mitigation of CH4 and CO2 emissions and blood profiles using crossbreed rams. J Sci Food Agric.

[13] Warner ACI (1964). The breakdown of asparagine, glutamine, and other amides by microorganisms from the sheep’s rumen. Aust J Biol Sci.

[14] Hoshino S, Sarumaru K, Morimoto K (1966). Ammonia anabolism in ruminants. J Dairy Sci.

[15] Chalupa W, Clark J, Opliger P, Lavker R (1970). Ammonia metabolism in rumen bacteria and mucosa from sheep fed soy protein or urea. J Nutr.

[16] Moss AR, Jouany JP, Newbold J (2000). Methane production by ruminants: Its contribution to global warming. Ann Zootech.

[17] Apajalahti J, Vienola K, Raatikainen K, Holder V, Moran CA (2019). Conversion of branched-chain amino acids to corresponding isoacids - an in vitro tool for estimating ruminal protein degradability. Front Vet Sci.

[18] Zhang HL, Chen Y, Xu XL, Yang YX (2013). Effects of branched-chain amino acids on in vitro ruminal fermentation of wheat straw. Asian-Australas J Anim Sci.

[19] McDougall EI (1948). Studies on ruminant saliva. 1. The composition and output of sheep’s saliva. Biochem J.

[20] Chaney AL, Marbach EP (1962). Modified reagents for determination of urea and ammonia. Clin Chem.

[21] Erwin ES, Marco GJ, Emery EM (1961). Volatile fatty acid analyses of blood and rumen fluid by gas chromatography. J Dairy Sci.

[22] Srinivas B, Gupta BN (1997). Rumen fermentation, bacterial and total volatile fatty acid (TVFA) production rates in cattle fed on urea-molasses-mineral block licks supplement. Anim Feed Sci Technol.

[23] Wang MZ, Wang HR, Cao HC, Li GX, Zhang J (2008). Effects of limiting amino acids on rumen fermentation and microbial community in vitro. Agric Sci China.

[24] Gilbreath KR, Nawaratna GI, Wickersham TA, Satterfield MC, Bazer FW, Wu G (2019). Ruminal microbes of adult steers do not degrade extracellular L-citrolline and have limited ability to metabolize extracellular L-glutamate. J Anim Sci.

[25] Sawers RG (2015). Amino acid degradation eLS.

[26] Argyle JL, Baldwin RL (1989). Effects of amino acids and peptides on rumen microbial growth yields. J Dairy Sci.

[27] López S, Dijkstra J, Forbes JM, France J (2005). In vitro and in situ techniques for estimating digestibility. Quantitative aspects of ruminant digestion and metabolism.

[28] Van Soest PJ (1994). Nutritional ecology of the ruminant.

[29] Anassori E, Dali-Naghadeh B, Pirmohammadi R, Hadian M (2015). Changes in blood profile in sheep receiving raw garlic, garlic oil or monensin. J Anim Physiol Anim Nutr.

[30] Janssen PH (2010). Influence of hydrogen on rumen methane formation and fermentation balances through microbial growth kinetics and fermentation thermodynamics. Anim Feed Sci Technol.

[31] Pengpeng W, Tan Z (2013). Ammonia assimilation in rumen bacteria: a review. Anim Biotechnol.

[32] Buckel W (2001). Usual enzymes involved in five pathways of glutamate fermentation. Appl Microbiol Biotechnol.

[33] Paster BJ, Russell JB, Yang CMJ, Chow JM, Woese CR, Tanner R (1993). Phylogeny of the ammonia-producing ruminal bacteria Peptostreptococcus anaerobius, Clostridium sticklandii, and Clostridium aminophilum sp. nov. Int J Syst Bacteriol.

[34] Newbold CJ, Lassalas B, Jouany JP (1995). The importance of methanogens associated with ciliate protozoa in ruminal methane production in vitro. Lett Appl Microbiol.

[35] Kingston-Smith AH, Davies TE, Edwards J, Gay A, Mur LAJ (2012). Evidence of a role for foliar salicylic acid in regulating the rate of post-ingestive protein breakdown in ruminants and contributing to landscape pollution. J Exp Bot.

[36] Russell JB, Mantovani HC (2002). The bacteriocins of ruminal bacteria and their potential as an alternative to antibiotics. J Mol Microbiol Biotechnol.

[37] Megías MD, Hernández F, Madrid J, Martínez-Teruel A (2002). Feeding value, in vitro digestibility and in vitro gas production of different by-products for ruminant nutrition. J Sci Food Agric.

[38] Raab L, Cafantaris B, Jilg T, Menke K (1983). Rumen protein degradation and biosynthesis: 1. A new method for determination of protein degradation in rumen fluid in vitro. Br J Nutr.

[39] Stiles DA, Bartley EE, Meyer RM, Deyoe CW, Pfost HB (1970). Effect of expansion-processed mixture of grain and urea (Starea) on rumen metabolism in cattle and on urea toxicity. J Dairy Sci.

[40] Zain M, Sutardi T, Suryahadi, Ramli N (2008). Effect of defaunation and supplementation methionine hydroxy analogue and branched chain amino acid in growing sheep diet based on palm press fiber ammoniated. Pak J Nutr.

[41] Suryapratama W, Suhartati FM (2005). Effect of supplementation of branched chain fatty acid on colony of ruminal bacteria and cell of protozoa. Anim Prod.

[42] Yang CMJ (2002). Response of forage fiber degradation by ruminal microorganisms to branched-chain volatile fatty acids, amino acids, and dipeptides. J Dairy Sci.

[43] Chen G, Sniffen CJ, Russell JB (1988). Fermentation of peptides and amino acids by monensin sensitive ruminal Peptostreptococcus. Appl Environ Microbiol.

