# Supplementary material for: Effects of L-glutamine supplementation on degradation rate and rumen fermentation characteristics in vitro
Source: Anim Biosci. 2021 Sep 15;35(3):422–33. doi: 10.5713/ab.21.0279 (PMC8902227; doi:10.5713/ab.21.0279)
Supplement: Supplementary file 1 [file ab-21-0279-suppl.pdf]

Supplementary tables

Table 2-1. Chemical composition of VFA standard

Ingredient composition	Amount (mL)	mM
Acetate (C_2)	0.35	58.29
Propionate (C_3)	0.15	20.25
Isobutyrate (C_3)	0.05	5.68
Butyrate (C_4)	0.10	11.35
Isovalerate (C_5)	0.05	4.90
Lactate (C_3)	0.05	4.90
DH_2O	100	-

Table 2-2. Chemical composition of NDF solution

Ingredient composition	Amount (g/2L DH_2O)
$C_{12}H_{25}NaO_4S$ (Sodium lauryl sulfate)	60.00
$C_{10}H_{14}O_8N_2Na_2 \cdot 2H_2O$ (Disodium hydrogen EDTA)	37.22
$Na_2B_4O_7 \cdot 10H_2O$ (Borax)(Sodium borate decahydrate)	13.62
Na_2HPO_4 (Disodium hydrogen phosphate anhydrous)	9.12

Table 2-3. Chemical composition of ADF solution

Ingredient composition	Amount (g/2L DH_2O)
$C_2H_{12}BrN$ (Ethyl ammonium bromide)	40
1N H_2SO_4 (99% Sulfuric acid)	56.1 ml

Table 2-4. Chemical composition of sulfanilammide standard

Ingredient composition (%)	Amount
Carbon (C)	41.84%
Hydrogen (H)	4.68%
Nitrogen (N)	16.27%
Oxygen (O)	18.58%
Sulfur (S)	18.62%
